# Headache and GLP-1 receptor agonists: when medications are therapeutic and when they contribute to the symptom

**DOI:** 10.1055/s-0045-1812303

**Published:** 2025-10-27

**Authors:** Erika Tavares Ferreira, Leidys Marina Pedrozo Garcia, Renata Gomes Londero

**Affiliations:** 1 Consultório privado, Jaraguá do Sul SC, Brazil.; 2 Consultorio privado, Valledupar, Cesar, Colombia.; 3 Hospital de Clínicas de Porto Alegre, Serviço de Neurologia, Porto Alegre RS, Brazil.

**Keywords:** Glucagon-Like Peptide-1 Receptor Agonists, Headache Disorders, Obesity, Migraine Disorders

## Abstract

Obesity is a complex metabolic disorder with significant implications for both individual and public health. It has been strongly linked to chronic headache conditions, including migraines and idiopathic intracranial hypertension (IIH). Individuals with obesity who suffer from migraine are at increased risk of chronification, while weight reduction has been associated with improvement in IIH-related headaches, likely due to a decrease in cerebrospinal fluid pressure. These observations underscore the importance of weight management strategies as a therapeutic consideration in patients with obesity and headache disorders. Glucagon-like peptide-1 receptor agonists (GLP-1 RAs) are pharmacological agents that mimic the hormone's endogenous activity. Analysis of selected studies highlights that these agents have emerged as a promising therapeutic option. The aim of this narrative review is to examine the role of GLP-1 RAs in the management of headaches, particularly in the context of IIH, migraine, and the gut–brain axis. Additionally, this review addresses the challenges associated with the use of this pharmaceutical class, including the potential for headaches as adverse effect, and identifies existing knowledge gaps that may guide future research.

## INTRODUCTION

Obesity is a condition associated with significant personal and social burden. 1 According to the World Health Organization (WHO), a body mass index ≥ 30 kg/m ^2^ defines obesity. Since the 1990s, the global prevalence of obesity has risen alarmingly, affecting both developed and developing countries. 2 In addition to being a major risk factor for cardiovascular disease, type-2 diabetes mellitus, and cancer, obesity also increases the risk of chronic pain syndromes, including headache disorders. 3 4

Glucagon-like peptide-1 (GLP-1) is an incretin hormone secreted by intestinal L-cells in response to nutrient intake. 5 Its primary role is to stimulate insulin secretion in a glucose-dependent manner and to suppress glucagon release, thereby contributing to glucose homeostasis. 6 Additionally, this hormone delays gastric emptying, enhances satiety, and reduces appetite, making it a key target in pharmacological strategies for weight management. 7 Glucagon-like peptide-1 receptor agonists (GLP-1 RAs) are synthetic agents that mimic the effects of the endogenous action of GLP-1 but offer enhanced stability due to resistance to enzymatic degradation by dipeptidyl-peptidase-4 (DPP-4). 8

Over the past decade, GLP-1 RAs have emerged as a promising class of therapeutic agents, initially recognized for their role in glycemic control in type 2 diabetes mellitus. 2 Notably, agents such as liraglutide, semaglutide, exenatide, and tirzepatide have also been employed in the management of obesity, not only promoting weight loss but also offering beneficial metabolic effects on comorbid conditions such as hypertension and dyslipidemia. 2

More recently, a growing body of evidence has indicated that therapeutic potential of GLP-1 RAs extends well beyond metabolic regulation, encompassing a range of actions within the central nervous system (CNS). 9 Studies have highlighted their capacity to modulate neuroinflammation and confer neuroprotective effects, raising intriguing possibilities for their use in related disorders. 7

In particular, weight loss induced by GLP-1 RAs has been associated with significant improvement in debilitating headaches, especially in conditions such as idiopathic intracranial hypertension (IIH) and migraine, both of which are known to be exacerbated by obesity and its associated systemic inflammation. 10 Furthermore, the anti-inflammatory properties and cytokine-modulating effects of GLP-1 RAs appear to contribute to pain regulation, implying a supplementary therapeutic modality. 11

The purpose of this paper is to provide a narrative review to explore the impact of GLP-1 RAs on headache management. We will examine their mechanisms of action within the CNS, their influence on inflammation and neuroprotection, and their potential to support weight reduction in the context of IIH and migraines. Additionally, we will consider their effects on the gut–brain axis, a growing area of interest in headache pathophysiology. Finally, this review will also address current challenges related to the use of GLP-1 RAs, such as the paradoxical occurrence of headaches as a side effect and highlight existing knowledge gaps to inform future research directions.

## METHODS

A comprehensive literature review was conducted using peer-reviewed articles published in English with no restrictions on publications date. The databases searched included PubMed, Embase, OVID, CINAHL, and Cochrane. The search strategy employed the following descriptors: (( *incretin mimetics* ) OR ( *glucagon-like peptide-1 receptor* ) OR ( *lose weight* )) AND (( *migraine* ) OR ( *headache syndromes* ) OR ( *intracranial idiopathic hypertension* )).

**Figure 1:**
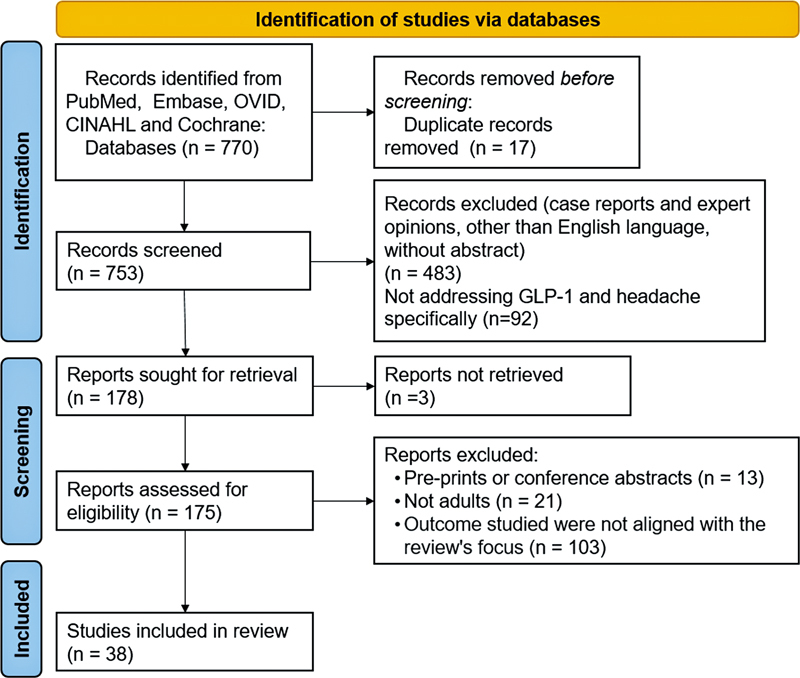
Preferred Reporting Items for Systematic reviews and Meta-Analyses (PRISMA) flow diagram for headache and GLP1 receptor: When medications are therapeutic and when they contribute to the symptom. **Source:** Page et al.

Priority was given to original research articles, randomized controlled trials (RCTs), and systematic reviews. Case reports and expert opinion pieces were initially excluded, to maintain a higher level of evidence. A total of 770 articles were identified through the initial search. After screening and excluding studies that did not specifically address relationship between GLP-1 RAs and headache disorders, 178 were retained for further evaluation. Of these, 38 were selected and included in the final analysis presented in this manuscript (Supplementary Material Table S1–available at https://www.arquivosdeneuropsiquiatria.org/wp-content/uploads/2025/08/ANP-2025.0045-Supplementary-Material.docx ) ( Figure 1 ).

## RESULTS

The analysis of the selected studies emphasized that GLP-1 RAs have an impact on headache management and other neurological conditions. Their metabolic and neurological effects synergistically contribute to neuroprotection. 7 While these drugs offer clear therapeutic benefits, they may also induce adverse effects in certain cases, 11 as shown in Figure 2 .

**NoteFigure 2:**
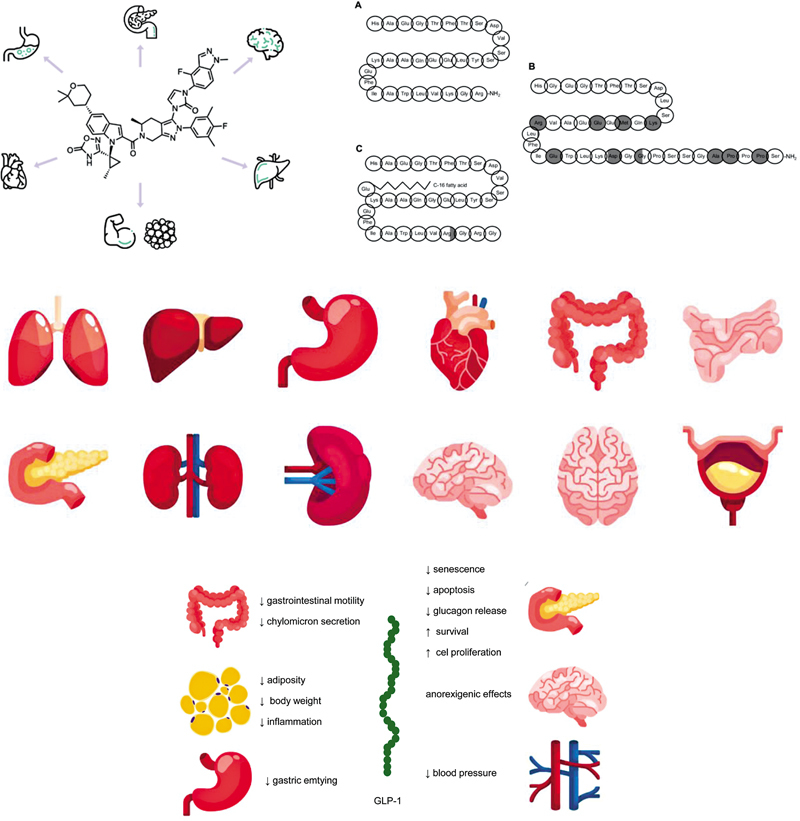
: License: Creative Commons Attribution-Noncommercial 3.0 Unported. The multisystem actions of glucagon-like peptide-1 (GLP-1) and its receptor agonists (GLP-1 Ras). ( **A** ) The molecular structure of human GLP-1. ( **B** ) The molecular structure of exenatide (gray colors indicate differences in structure from human GLP-1. ( **C** ) The molecular structure of liraglutide (gray colors indicate changes in structure from human GLP-1).

The activation of GLP-1 receptors (GLP-1 Rs) in the CNS plays a crucial role in modulating neuroinflammation and providing neuroprotection. In the hypothalamus and brainstem, receptor activation initiates signaling pathways that support cell growth and survival, enhance synaptogenesis, and stimulates autophagy, which is essential for clearing damaged proteins and maintaining cellular homeostasis. 12 Furthermore, GLP-1RAs reduce the secretion of proinflammatory cytokines, namely interleukin 6 (IL-6) and tumor necrosis factor alpha (TNF-α), which are often elevated in neuroinflammatory conditions like migraine. 1 By preventing apoptosis, fostering synaptic regeneration, and supporting neuronal recovery, GLP-1 RAs may contribute to clinical improvement across a range of neurological disorders. 3 4

Obesity is a major modifiable risk factor for chronic migraine, contributing to central sensitization and exacerbating the frequency and severity of attacks. 3 4 Weight loss influences headache patterns in certain patients. During digestion, intestinal peptides such as GLP-1 and amylin are released and delay gastric emptying. 13 Concurrently, glucagon, secreted by the pancreas, and gastric leptin modulate appetite and reduce food intake by signaling satiety to the brain. 14

The gut–brain axis is regulated by a dynamic balance between proinflammatory influences (such as sugar-rich diets that increase cell oxidative stress) and anti-inflammatory factors (such as a healthy diet, stress management, regular physical activity). 15 This equilibrium can be disrupted by factors that promote chronic low-grade inflammation, including sedentary behavior, exposure to xenobiotics, environmental pollution, inadequate nutrition, and sleep disturbances. 16 These factors collectively contribute to the development of neurodegenerative diseases, depression, cancer, and intestinal dysbiosis. 1 Over time, dysbiosis may aggravate conditions driven by neurogenic inflammation, such as migraines. 9

The homeostasis of the gut–brain axis can be altered in other pathologies, including IIH. 5 In women, an increase of more than 20% in body weight increases the incidence of IIH from 0.9 to 19.3 cases per 100 thousand inhabitants. 15 Researchers have recently discovered the remarkable benefits of GLP-1 RAs in individuals with IIH. 17

As a neurological disorder, IIH is characterized by elevated cerebrospinal fluid (CSF) pressure, which can lead to severe headaches, visual field disturbances due to papilledema, and ocular motor nerves palsy, among other features. 2 The condition has an incidence of 0.9 per 100 thousand people, exhibits a striking 8:1 female preponderance, and is frequently associated with obesity. 7 A reduction in body weight of just 5 to 10% has been shown to lower CSF pressure, which is directly correlated with headaches in IIH. 8 Consequently, pharmacologic agents such as liraglutide and semaglutide, which induce weight loss and reduce intracranial pressure, may provide substantial relief from IIH symptoms, significantly enhancing patients' quality of life. 18 However, long-term weight maintenance and adherence to treatment remain critical to achieving and sustaining clinical improvement. 5

The link between obesity and IIH is well established, with weight loss resulting in measurable symptom relief. 19 Obesity is hypothesized to raise intracranial pressure via increased intra-abdominal pressure, which elevates venous pressure. 20 Additionally, estrogen-mediated pelvic adiposity in women leads to a prothrombotic state and further weight gain. 12 Adipose tissue expansion increases leptin levels, which regulate appetite, energy expenditure, and glucose and lipid metabolism via hypothalamic signaling. 19 In this context, reducing body mass helps mitigate the chronic low-grade inflammatory state that underpants IIH pathogenesis. 21 Notably, GLP-1 promotes weight loss through both neuronal and metabolic mechanisms. 11 It is present in the human choroid plexus, where GLP-1 RAs reduce CSF secretion and intracranial pressure by elevating intracellular cyclic adenosine monophosphate levels and inhibiting the Na +/K+ ATPase pump. 22

Beyond their utility in IIH, GLP-1 RAs play a role in migraine management. 23 Preclinical studies have demonstrated that liraglutide stimulates IL-10 secretion, an anti-inflammatory cytokine, thereby alleviating migraine-like pain in rats. 24 Activated GLP-1 R also suppresses the production of pro-inflammatory cytokines such as interleukin 1 beta (IL-1β) and TNF-α, which are upregulated during migraine attacks. 7 Furthermore, these RAs modulate microglial activity, helping reduce chronic inflammation that contributes to central sensitization and symptom persistence. 12 The central mechanisms involved in migraine attenuation include restoration of immune cell balance and activation of neuroprotective pathways. 25

Despite the benefits, some patients report increased headache frequency or intensity after initiating GLP-1 RA therapy. 11 This adverse effect may be related to GLP-1 R activation in the CNS, which can influence cerebrovascular tone and regional blood flow, potentially resulting in cerebral vasodilatation and headaches due to mechanical strain on pain-sensitive structures. 26 27 Additionally, GLP-1-mediated modulation of pain pathways, particularly via microglial activation in the caudal trigeminal nucleus, may exacerbate central sensitization and worsen headache symptoms. 12 This phenomenon reflects the dynamic neurohormonal and metabolic shifts provoked by GLP-1 RAs therapy. 13 Headache is most often reported during the early stages of treatment and is usually transient. 13

A meta-analysis by Filippatos et al. reported that, since 2009, headaches have been documented across several GLP-1 RAs trials. 26 They are generally mild, nondisabling, and most frequently occur within the first 26 weeks of treatment. They have been observed with liraglutide (1.8 mg daily), and semaglutide (1.5 mg weekly), exenatide (2 mg weekly), and dulaglutide (0.75 to 1.5 mg weekly). 28 29 30 Importantly, these events rarely lead to treatment discontinuation, unlike more severe adverse effects, such as pancreatitis, hypoglycemia, or dermatological reactions, which may necessitate withdrawal due a life-threatening risk. 31

Ongoing research aims to identify biomarkers that may predict susceptibility to GLP-1 RA-induced headaches. Such predictive tools would support personalized therapy and optimize patient outcomes. 13 17 It is essential to recognize that most individuals undergoing this treatment are obese, and that primary headaches are more prevalent and disabling in this population, with migraine being the most common diagnosis. 32 Obesity increases the risk of both episodic and chronic migraine through bidirectional central and peripheral mechanisms. 20 Nonetheless, headache occurring during GLP-1 RA therapy may not be directly drug-induced, as secondary factors like dehydration and hypoglycemia may contribute. 3 4 Vomiting and nausea, common gastrointestinal side effects, can lead to dehydration, which is the predominant non-neurological contributor to headaches during treatment. 26

Fasting-related headaches also merit consideration. These typically result from prolonged fasting, caloric restriction, dehydration, or hypoglycemia. 6 When this last one is the cause, the headache often arises after at least eight hours without food and is relieved by eating. 6 33 Clinically, it presents as a widespread pain, mild-to-moderate and nonpulsatile, in the frontal region. 23 Although hypoglycemia is uncommon with GLP-1 RAs alone, the risk increases when used in combination with insulin or sulphonylureas. Therefore, patients should avoid extended fasting while on these medications. 26

## DISCUSSION

The use of weight loss medications, particularly GLP-1 RAs, is increasing steadily, both with and without medical supervision. Consequently, clinicians are more frequently confronted with the context of headache disorders. 17 These medications are often highly regarded by patients for their striking benefits, not only in terms of weight reduction but also of improving associated comorbidities such as hypertension, diabetes, depression, and anxiety. 11 This raises a timely clinical question: Could GLP-1 RAs represent a therapeutic strategy for obese patients with migraine or other headaches who also struggle with weight management? 22

To make a well-informed, individualized decision, it is essential to recognize the variability in both therapeutic benefits and adverse effects among patients treated with GLP-1 RAs. 2 Factors such as a metabolic phenotype, presence of comorbidities, and individual sensitivity to hormonal fluctuations and weight loss play a critical role in treatment response. 11 Furthermore, treatment duration and dosage significantly influence both efficacy and tolerability. These variables underscore the importance of prescribing GLP-1 RAs under the guidance of experienced physicians, a message that must be clearly communicated to patients. 34

While most headache exacerbations are observed in patients with a prior history of migraine, new onset (de novo) headaches have also been reported during GLP-1 RA therapy. A pharmacovigilance analysis using data from the FDA's Adverse Event Reporting System identified a statistically significant association between GLP-1 RAs and the onset of headache, with a reporting odds ratio (ROR) of 1.74, and migraine (ROR: 1.28), suggesting that these symptoms can develop regardless of pre-existing headache diagnoses. 17 35 Therefore, careful clinical monitoring is warranted, especially during the early phases of treatment.

Another critical consideration is the role of obesity itself as a risk factor and disease modifier in migraine. It has been associated with increased migraine frequency, severity, and risk of chronification. 10 Weight loss reduces systemic inflammation and mitigates metabolic contributors, such as insulin resistance, oxidative stress, and cerebrovascular dysregulation. 27 Thus, the GLP-1 RAs directly exert dual benefits: through their anti-inflammatory and neuroprotective mechanisms, and by improving metabolic variables that worsen headache burden. 25

Finally, emerging research is exploring how GLP-1 RAs may modulate central pain pathways, an area with significant therapeutic promise. 17 These findings may pave the way for novel interventions targeting chronic headache and migraine, particularly in patients with treatment-resistant forms of the disease. If substantiated by further clinical trials, such approaches could meaningfully improve the quality of life in this population, highlighting the need for continued, rigorous investigation. 24

In conclusion, GLP-1 RAs have emerged as promising therapeutic agents for neurological conditions, with underlying metabolic and inflammatory components, including headache disorders in obese individuals. 25

Given the notable interindividual variability in both efficacy and tolerability, a personalized prescription approach is essential to optimize outcomes and minimize adverse effects. 36 Future efforts to identify predictive biomarkers, which are more likely to benefit from GLP-1 RAs, will be key to stratifying patients based on likely therapeutic response or risk of side effects. 9

As this field evolves, clinical observations and patient-reported outcomes should be more fully integrated into the research process. These perspectives are not anecdotal; rather, they offer valuable insight into the real-world impact of treatment and can help refine hypotheses about the pathophysiology of migraine. 17 A more collaborative interface between research and clinical practice will be essential in addressing the complex and multifactorial nature of headache disorders.

Emerging evidence also underscores the involvement of metabolic pathways in migraine pathogenesis, revealing novel therapeutic targets. 37 Through their anti-inflammatory and neuromodulatory actions, GLP-1 RAs may offer an additional mechanism for migraine control, particularly in cases with obesity, as it is a known risk factor for chronification. To date, however, no studies have specifically assessed this endpoint in migraine populations, highlighting a critical gap in the literature and an opportunity for future clinical trials. 7

In combination with existing prophylactic treatments, 38 GLP-1 RAs may represent a valuable option for the subset of obese patients, especially those who are refractory to first- and second-line treatments, expanding the therapeutic armamentarium available to the neurologists managing these complex cases.
